# COVID-19 and Tuberculosis Co-infection in Pregnancy – a Case Series and Review

**DOI:** 10.34763/jmotherandchild.20212502.d-21-00002

**Published:** 2022-04-01

**Authors:** Pranav Modi, Roshni Khanna, Nanditha Reddy, Ashwini Patankar, Shahid Patel, Girija Nair, Sriram Gopal, Abhay Uppe

**Affiliations:** 1 Department of Pulmonary Medicine, D Y Patil Medical College and Hospital, Nerul, Navi Mumbai, India; 2 Department of Obstetrics and Gynaecology, D Y Patil Medical College and Hospital, Nerul, Navi Mumbai, India; 3 Department of Internal Medicine, D Y Patil Medical College and Hospital, Nerul, Navi Mumbai, India

**Keywords:** COVID-19 and TB co-infection, COVID-19, TB, COVID-19 and TB co-infection in pregnancy, pregnancy, COVID-19 in pregnancy, TB in pregnancy

## Abstract

Various guidelines are in place for management for COVID-19 and pulmonary tuberculosis (PTB) in pregnancy. However, to the best of our knowledge, there are no significant guidelines for the management of COVID-19 and PTB co-infection in pregnancy. Pregnancy being an altered physiological state, the use of various drugs and their outcomes are altered. Here we present two cases of COVID-19 and PTB co-infection in pregnancy which were managed successfully.

## Introduction

The COVID-19 pandemic has had a major impact on healthcare delivery in India and worldwide. Knowledge about COVID-19 management is still evolving, and the risk of infection and its impact on health are being extensively studied in the population. However, the risks to foetus due to COVID-19 during pregnancy are not fully known, and co-infection with pulmonary tuberculosis (PTB) can further complicate the situation. Guidelines for management of PTB in pregnancy have been well established. The CDC suggests that antitubercular treatment should be started early when the probability of TB is moderate to high, since untreated disease presents a greater hazard to mother and foetus than when treated. Various regimens for management of latent tuberculosis and active tuberculosis include daily and weekly regimens lasting up to 9 months. Guidelines for drug-resistant TB and drugs to avoid in pregnancy are also in place. [1] Current Indian guidelines under the National

Tuberculosis Elimination Program (NTEP), as well as the World Health Organization (WHO), support the use of standard treatment regimen in pregnancy. This regimen consists of isoniazid (H), rifampin (R), pyrazinamide (Z) and ethambutol (E) (HRZE) for 2 months (intensive phase), followed by HRE for 4 months (continuation phase). Despite their ability to cross the placenta, antitubercular drugs have no harmful effects on the foetus. The dosage and duration of therapy need no modification in pregnancy. [2] Pregnancy in the setting of COVID-19 has been well described. Pregnant women are at no added risk of contracting the disease. However, the Royal College of Obstetricians and Gynaecologists (RCOG), Federation of Obstetric and Gynaecological Societies of India (FOGSI) and Centre for Disease Control and Prevention (CDC), all regard pregnancy to be a state of moderate risk. [3]

Hence, social distancing should be meticulously practised during pregnancy, especially in the third trimester. Pregnant women over the age of 34, with or without comorbidities, were found to have a higher risk of developing pulmonary complications. [3] This is because pregnancy alters the general physical state of the woman and is an immunosuppressive condition, causing certain viral infections which may result in worse outcomes in pregnancy. Furthermore, the pregnancy bias towards T-helper 2 (Th2) system dominance, which protects the foetus, leaves the mother vulnerable to viral infections, which are contained by the Th1 system. [4]

Guidelines suggested by Indian Council of Medical Research (ICMR) take this into account, thereby reducing exposure by those infected to the general population by reducing antenatal visits, routine ultrasounds, provision of isolation rooms, separation of infants from mothers and training of health personnel for practicing personal protection. [5] It also covers reduction of risk to the pregnant woman by decreasing incidence of unindicated surgical interventions, use of instrument vaginal delivery where possible, continuous monitoring of vitals of both mother and foetus and delivery at a tertiary care centre with easy availability of anaesthesia, operative interventions and intensive care units (ICU). [6]

Various case reports, descriptive studies and longitudinal analyses suggest that the majority of pregnant women admitted with COVID-19 infection were in the third trimester of pregnancy. [7] For asymptomatic and mild COVID-19 infections in pregnancy, the management of labour is not altered. [8] However, C-section was the preferred mode of delivery due to the significant risk of postpartum haemorrhage and premature delivery. [7] COVID-19 leads to a procoagulant state called COVID-19-associated coagulopathy (CAC). It has been associated with an increased mortality, and hence the use of Low molecular weight heparin (LMWH) can be considered for thromboprophylaxis in pregnancy. [9] Among newborns, despite the lack of concrete evidence of vertical transmission, cases with mild pneumonia were reported but recovered. [7]

Recently, the Ministry of Health and Family Welfare (MOHFW), India has described a bidirectional TB-COVID screening strategy that proposes COVID-19 screening for all diagnosed TB patients and vice versa. [10] However, the diagnosis and specific management of TB-COVID co-infection in pregnancy have not been described. Hence, it is imperative that we study these aspects further, classify the severity and take appropriate management decisions. We present a series of two cases from a tertiary care hospital in Navi Mumbai, India, with TB-COVID co-infection in pregnancy and discuss its management.

## Review

### Case 1

A 26-year-old married female, G2P1L0D1 at 26 weeks of pregnancy presented with an expectorant cough for 4 months and sore throat for 2 months. She complained of dyspnoea for 1 month (Grade 2 MMRC). The patient also complained of an evening rise of temperature and four episodes of moderate haemoptysis.

The patient’s first pregnancy was terminated in view of eclampsia by a full term LSCS (lower segment Caesarean section) delivering a baby girl of 2.5 kg that died 2 years ago (infant death of unknown cause). There were no other known comorbidities.

COVID-19 RT-PCR was positive on day 2 of admission after testing negative twice earlier (2 months before admission, RT-PCR was carried out as a routine check-up). Chest X-ray with abdominal shield revealed bilateral inhomogeneous infiltrates and a thick-walled cavity with air-fluid level in the lower zone of the right lung field. The patient was diagnosed with sputum-positive TB status with sputum Gene-Xpert (cartridge-based nucleic acid amplification test (CBNAAT) positive for *Mycobacterium tuberculosis* without rifampicin resistance on day 3. For the first 3 days, the patient was managed in the isolation ward, after which she was shifted to ICU in view of tachypnoea and tachycardia with desaturation. Hemodynamic parameters revealed blood pressure of 90/50 mm Hg and a pulse of 126 beats/min with a respiratory rate of 48 cycles/min. On auscultation, breath sounds were reduced in the right-side inframammary region with the presence of rhonchi. Oxygen support was provided at 15 L/min along with intermittent non-invasive ventilation (NIV). Arterial blood gases were monitored every 12 h. IV fluids and noradrenaline infusion (8 mcg/50 mL) at 2.5 mL/h were immediately started. Haemoptysis was controlled with injectable etamsylate (250 mg) and tranexamic acid (500 mg).

Under the cover of broad-spectrum antibiotics, the patient was started on standard anti-tubercular drugs under directly observed treatment, short-course (DOTS) according to the weight regimen, with a protein rich diet. Due to co-infection with COVID-19, injectable remdesivir was started for use on compassionate grounds with supportive management as per standard protocol. The use of remdesivir was justified on grounds of benefits outweighing risks and was tolerated well alongside antitubercular therapy. After six doses of remdesivir and 10 days of intensive care, the oxygen requirement reduced to 1–2 L through nasal prongs with normal haemodynamic parameters and no further episodes of haemoptysis were demonstrated. The patient was stepped down to the COVID ward and discharged for home isolation with pulmonary medicine and OB-GYN follow up after 2 weeks.

### Case 2

A 27-year-old married female, primigravida, at 38 weeks of pregnancy presented with a dry cough for 2 months and dyspnoea for 2 days (Grade 1 MMRC). The patient also had fever for 3 days associated with generalised weakness and tested positive for COVID-19 (RT-PCR). Decision for elective LSCS was made in view of oligohydramnios with amniotic fluid index (AFI) 0–1 on USG. The patient was hemodynamically stable with a pulse rate of 104 beats/min, a respiratory rate of 22 cycles/min, blood pressure of 130/70 mm Hg and oxygen saturation of 96% on room air. A healthy baby girl was delivered weighing 2890 g. Post-delivery, HRCT (high resolution computed tomography) thorax was done, was and the results were suggestive of multiple areas of ground glass opacities in bilateral lung fields with a CT-severity score of 10/25 and CORADS 6. Consolidation with air bronchogram and a cavity showing air crescent sign were visible in the left upper lobe. Sputum samples were negative for culture and sensitivity, fungal stain, AFB smear and Gene-Xpert. The patient was further managed in ICU where oxygen support was provided at 2 L/min through nasal prongs, and bronchoscopy was done. Bronchoalveolar lavage (BAL) samples were sent for culture and sensitivity, fungal stain, AFB smear, cytology, Gene-Xpert, TB-MGIT and fungal culture and sensitivity. BAL Gene-Xpert returned positive for *Mycobacterium tuberculosis* with no rifampicin resistance. For COVID-19, injectable remdesivir was given for the next 5 days on compassionate grounds. The patient was started on standard antitubercular therapy under DOTS on a weight-based regimen with a protein rich diet. Prophylactic antitubercular regimen was started for the newborn child as well. BAL-culture was positive for *Klebsiella pneumonie* that was treated with amoxicillin–clavulanic acid for 10 days based on the drug sensitivity report. The patient was shifted to COVID ward after completing 5 days of ICU stay and was discharged for home isolation and self-monitoring. Patient was asked to follow up with OB-GYN and pulmonary medicine after a week.

## Discussion

Physiological changes that occur in pregnancy may exacerbate the effects and changes caused by the *SarsCov-2* virus. The virus enters the body through the ACE-2 receptors that are up-regulated in pregnancy. Due to this up-regulation, pregnant women are at a higher risk of contracting severe acute respiratory infection due to COVID-19. Upon binding to ACE-2 receptors, *SarsCov-2* causes its down-regulation, consequently leading to increased vasoconstriction and risk of developing preeclampsia. [11] In a recent report from the US Center for Disease Control and Prevention (CDC), pregnant patients had higher rates of ICU admission, invasive ventilation and death as compared to non-pregnant patients with confirmed COVID-19 infection. [12] Another report by the CDC suggested that pregnant women with confirmed COVID-19 who were hospitalised had higher rates of preterm birth and C-section delivery. [11,13]

The use of ionising radiation in pregnant women is subject to defined constraints. As a result, CT scan that is indispensable in the diagnosis and extent of COVID-19 damage, is contraindicated in pregnancy. There is a need to explain risks to the mother and also explain benefits for it. The maximum permitted dose of radiation exposure is below 50 mGy in pregnant women, which is essentially safe for the foetus. When chest CTs and chest X-rays are indicated, local protection for the foetus (abdominal lead shields) must be utilised, especially in the first trimester. One approach is to get a chest X-ray and proceed to CT only if abnormal. [14]

Most cases of COVID-19 in pregnancy are diagnosed in the third trimester and there may be a risk of iatrogenic PTB in view of deterioration of mother’s health with COVID-19 complications. [15]

With regard to the health of the foetus, an increased incidence of IUGR and loss of foetal wellbeing may eventuate due to COVID-19 in pregnancy. Those with existing comorbidities are at a higher risk of these complications. However, spread of the disease has not been documented through vertical transmission or through breast milk, amniotic fluid or vaginal secretions. There is also negligible chance of passage of virus through the placenta. [16]

Pregnant women should follow similar strategies to avoid exposure to *SarsCov-2* as compared to non-pregnant women. However, this is applicable only if symptomatic, pregnant women present in a similar way as compared to the non-pregnant women. The ACOG/SMFM (American College of Obstetricians and Gynecologists - ACOG, the Society for Maternal-Fetal Medicine - SMFM) have developed an algorithm to aid practitioners to evaluate and treat pregnant women with known exposure and/or those with symptoms consistent with COVID-19 (persons under investigation [PUI]) that categorises them into low-, moderate- and high-risk categories. [17]

Co-infection with tuberculosis in this scenario can further complicate matters since it presents with similar symptoms though with a longer incubation period. Tuberculosis was found to be prevalent in 0.37–4.47% of COVID-19 patients, and those with active tuberculosis infection have more than twice the risk of contracting severe COVID-19 infection. The MOHFW, India provides the following guidance regarding screening of COVID-19 and TB co-infection. [10]

A bidirectional TB-COVID screening strategy promotes COVID-19 testing for all diagnosed TB patients and TB screening for all COVID positive patients. For those with confirmed COVID-19 and presenting with constitutional symptoms of tuberculosis, in close contact with a TB case and past history of PTB, chest X-ray and sputum Nucleic Acid Amplification Test (NAAT) – CBNAAT/TrueNat) should be offered. [10] However, due to imaging constraints in pregnancy, efforts should be made to minimise exposure to ionising radiation, and we recommend that chest X-ray or CT thorax should be considered only according to the risk versus benefit for the given clinical scenario. [18] With imaging now considered to be of prime importance in diagnosis and management of COVID-19, the Iranian Society of Radiology has devised guidelines for the use of imaging techniques with optimal protection and safety for COVID-19 confirmed/suspected pregnant women. [14] As per the guidelines, it would be prudent to avoid imaging in pregnant women suspected to have COVID-19. If unavoidable and strongly indicated, chest X-ray or CT scans should be done only after obtaining informed consent and explaining the potential risks and benefits of the test to the patient. Also, an abdominal shield must be used if imaging is indicated in the first trimester. A low-dose CT may be done in the second and third trimester if chest X-ray is inconclusive. [14]

Both patients in our series presented in late pregnancy and underwent sputum testing for AFB and Nucleic Acid Amplification Test (NAAT) along with imaging as indicated. For Case 1, a chest X-ray was done using an abdominal shield, and for Case 2, HRCT thorax was done prior to bronchoscopy.

The antenatal, intrapartum and postnatal care for pregnancy with COVID-19 infection is illustrated in Figures 3, 4, 5. [16,19]

**Figure 1:**
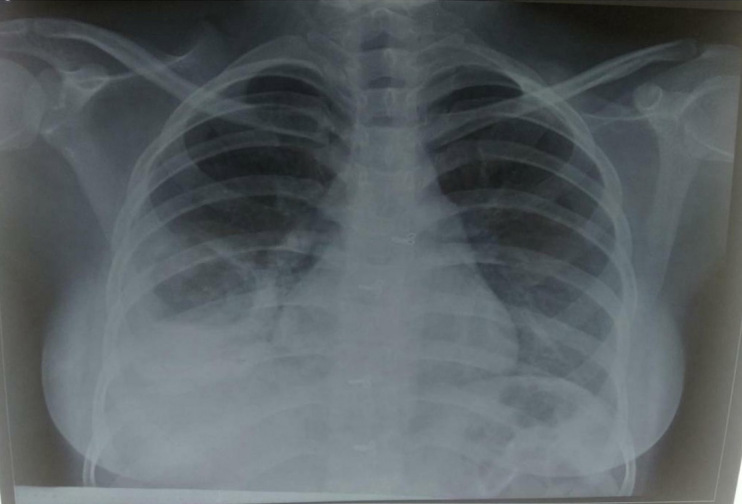
Chest X-Ray with abdominal shield suggestive of bilateral inhomogeneous infiltrates and a thick-walled cavity with air-fluid level in the lower zone of the right lung field.

**Figure 2:**
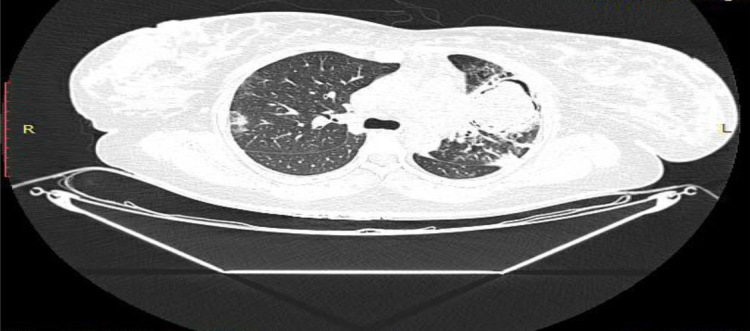
HRCT thorax suggestive of consolidation with air bronchogram and a cavity showing air crescent sign in the left upper lobe. Multiple areas of ground glass opacities were present in bilateral lung fields with a CT-severity score of 10/25 and CORADS 6.

**Figure 3:**
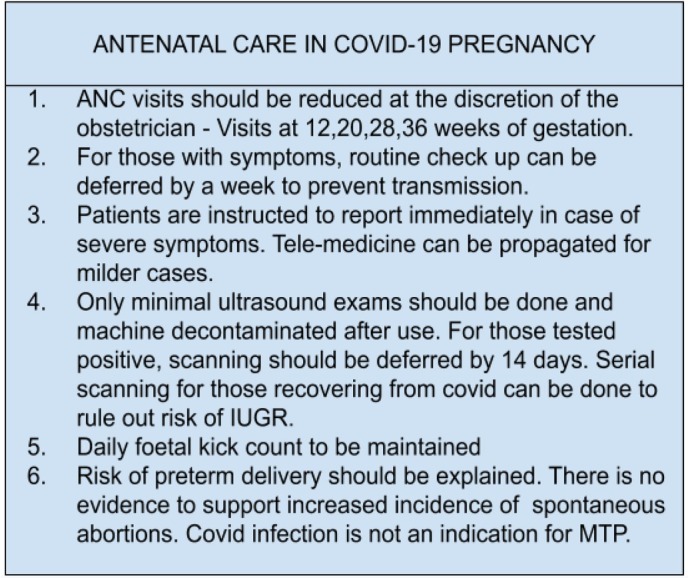
Antenatal Care in COVID-19 pregnancy

**Figure 4:**
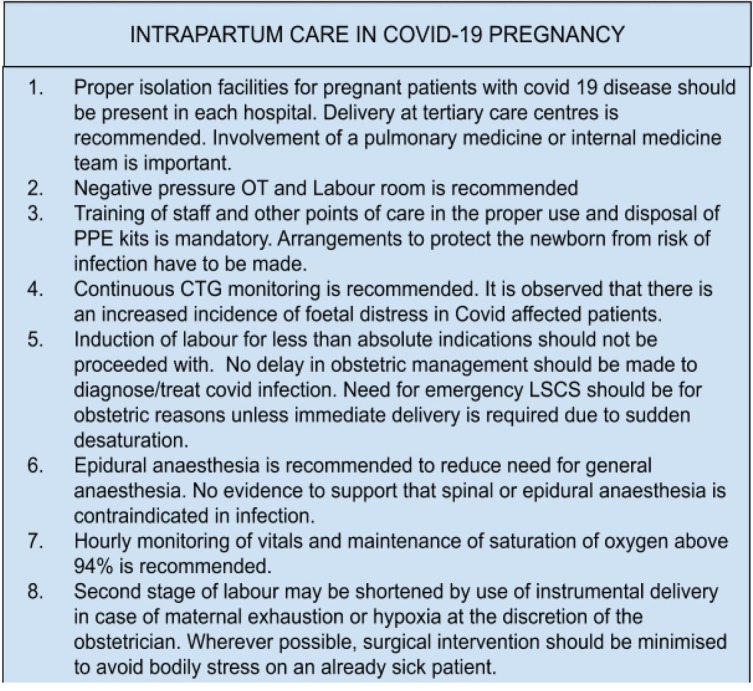
Intrapartum care in COVID-19 pregnancy

**Figure 5:**
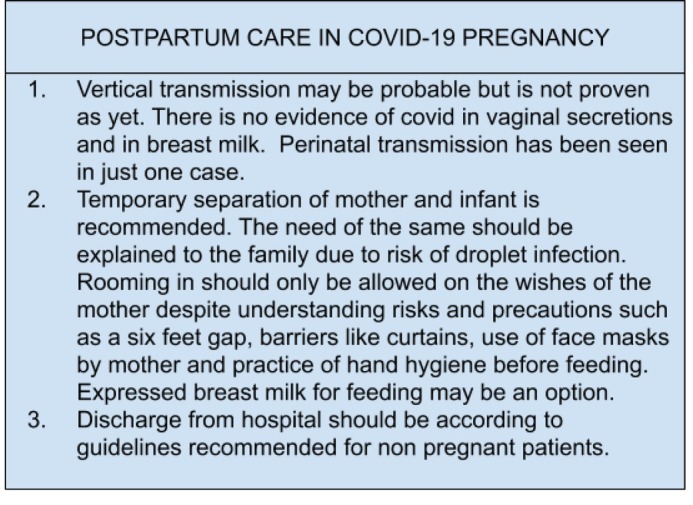
Postpartum care in COVID-19 pregnancy

**Figure 6:**
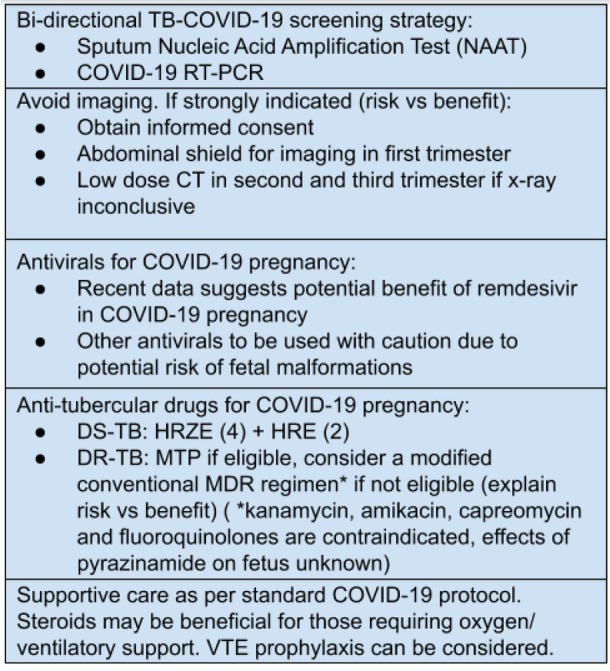
Management of TB-COVID co-infection in pregnancy

Data regarding the treatment for COVID-19 in pregnant women are limited, and most trials evaluating the use of the antiviral remdesivir have excluded pregnant women. Results from a compassionate-use programme involving 86 pregnant women with confirmed COVID-19 infection who received 10 doses of injectable remdesivir were suggestive of high recovery rates and a low rate of serious adverse effects. [19] Both patients in our series qualified for the use of injectable remdesivir. Other drugs like hydroxychloroquine should be used with caution as they pose a minor risk of foetal malformations during the first trimester. [20]

Guidelines have been formulated by the CDC for management of tuberculosis in pregnancy with regimens for latent TB infection, TB disease and HIV-related TB disease. For latent TB infection, the commonly used regimens are the either the 4-month daily regimen of rifampin (RIF) (4R), a 3-month daily regimen of isoniazid (INH) and RIF (3HR), 6- or 9-month daily regimen of INH (6H or 9H) with pyridoxine (vitamin B6) supplementation. For those with TB disease, initial treatment is with INH, RIF, and ethambutol (EMB) daily for 2 months, followed by INH and RIF daily, or twice weekly for 7 months (for a total of 9 months of treatment). Streptomycin should be avoided since it can cause harmful effects on the foetus. Pyrazinamide is not preferred since its effects on the foetus are unknown. Kanamycin, amikacin, capreomycin and fluoroquinolones are also contraindicated in pregnancy. [1]

For the Indian scenario, a collaborative framework for management of tuberculosis in pregnant women has been laid out by the MOHFW. During the ANC period, a standard treatment regimen must be followed for drug-sensitive TB. A 32 weeks USG focussing on diagnosis of foetal complications must be performed, as well. In the case of drug resistant TB, the patient should be advised for medical termination of pregnancy if the duration of pregnancy is less than 20 weeks. For those unwilling for MTP or not eligible for MTP (duration of pregnancy more than 20 weeks), a modified conventional MDR-TB regimen may be started, clearly explaining its risks to the mother and foetus. [2]

Both patients in our series were diagnosed as drug-sensitive TB and were started on a standard weight-based regimen under DOTS comprising HRZE for 2 months (intensive phase) followed by HRE for 4 months (continuation phase) along with vitamin B6 supplementation and a high protein diet.

Regarding steroid use in COVID-19 pregnancy, recent evidence supports the early use of short-course glucocorticoids for those patients requiring oxygen support or mechanical ventilation. [21] However, steroids were not given to both patients in our series in view of TB co-infection and decreasing oxygen requirement.

Supportive treatment for COVID-19 was provided as per standard protocol. Acetaminophen was preferred over NSAIDs in our series on an SOS basis for fever, based on limited evidence and anecdotal reports raising concerns for possible negative effects of NSAIDS in young non-pregnant COVID-19 patients. [22] Prophylactic dose of anticoagulation with unfractionated heparin was given as VTE prophylaxis with limited evidence suggesting the risk of developing VTE due to COVID-19 in pregnant females. [23]

Pregnant patients with COVID–TB coinfection must be closely monitored for complications such as acute respiratory failure, ARDS, pneumonia, sepsis due to COVID-19 and haemoptysis, pneumothorax, bronchiectasis and chronic pulmonary aspergillosis due to PTB.

## Conclusions

The treatment of COVID–TB coinfection can be challenging in pregnancy. Recent data support the use of remdesivir, and patients in our series tolerated the combination of antiviral and antitubercular treatment well. However, further randomised controlled trials are needed to establish the safety and efficacy of the same.

## Disclaimer

COVID-19 is an emerging, rapidly evolving situation and we recommend healthcare professionals to review the latest official guidelines from local governments and health organisations.
